# COVID-19 pandemic response in the Meuse-Rhine Euroregion: methods, participation and recommendations of a longitudinal cross-border study

**DOI:** 10.1186/s13690-023-01102-6

**Published:** 2023-05-13

**Authors:** C. Stabourlos, C. J. A. van Bilsen, S. Brinkhues, C. P. B. Moonen, S. Demarest, D. A. T. Hanssen, I. H. M. van Loo, P. H. M. Savelkoul, D. Philippsen, B. A. M. van der Zanden, N. H. T. M. Dukers-Muijrers, C. J. P. A. Hoebe

**Affiliations:** 1 Department of Epidemiology and Public Health, Sciensano, Rue Juliette Wytsmanstraat 14, Brussels, 1050 Belgium; 2 Department of Sexual Health, Infectious Diseases and Environmental Health, Living Lab Public Health, Public Health Service South Limburg, Heerlen, the Netherlands; 3 Department of Social Medicine, Care and Public Health Research Institute (CAPHRI), Maastricht University, Maastricht, The Netherlands; 4 Public Health Service South Limburg, Department of Knowledge and Innovation, Public Health Service (GGD) South Limburg, Heerlen, the Netherlands; 5 Department of Medical Microbiology, Care and Public Health Research Institute (CAPHRI), Maastricht University Medical Centre, Maastricht, The Netherlands; 6 GesundheitsberichterstattungGesundheitsamt Düren, Düren, Germany; 7 Foundation euPrevent, Heerlen, The Netherlands; 8 Department of Health Promotion, Care and Public Health Research Institute (CAPHRI), Maastricht University, Maastricht, The Netherlands

**Keywords:** Meuse-Rhine Euroregion, Health, Cross border, Prospective longitudinal studies, International health regulations, COVID-19 serological testing, SARS-CoV-2, Pandemic response, Pandemic preparedness

## Abstract

**Background:**

Comparative data collection in transborder areas can contribute to informed decision making processes when dealing with borderless health threats such as pandemics, and thus help minimize the negative health effects for its citizens. To examine the pandemic response over time and the impact of infectious disease control in a cross-border setting, a prospective longitudinal study was conducted in the border area between Germany, Belgium and the Netherlands. In the spring of 2021, a random sample of 26,925 adult citizens selected from governmental registries was invited to collect a blood sample at home for SARS-CoV-2 antibody testing and to fill in an online questionnaire on attitudes and behaviour towards infection prevention measures, cross-border mobility, social network and support, COVID-19 self-reported infection(s) and symptoms, vaccination, general self-reported health and socio-demographics. In autumn 2021, participants were invited for a follow-up round. An online tool was developed to coordinate fieldwork procedures, real-time monitoring of participation and consultation of antibody test results. Furthermore, a helpdesk in all three languages for participants’ support was set up.

**Results:**

In the first round, 6,006 citizens in the Meuse-Rhine Euroregion participated. 15.3% of the invited citizens on the Belgian side of the border participated. In the Netherlands and Germany this was respectively 27% and 23.7%. In the follow-up round 4,286 (71.4%) citizens participated for the second time. The participation rate was highest in the age group 50–69 years and lowest in > 80 in all sub regions of the Meuse-Rhine Euroregion. More women participated than men. Overall, more blood samples were returned than completed questionnaires. In total, 3,344 citizens in the Meuse-Rhine Euroregion completed all components of participation in both rounds.

**Conclusions:**

The collection of comparative data can help better assess the pandemic response and the impact of infectious disease control in a cross-border area. Recommendations for a longitudinal cross-border study include a centralized online environment, mapping out potential challenges related to national regulations in the preparation phase and organizing regional coordination centres to create more familiarity and trust towards the involved organisations.

**Supplementary Information:**

The online version contains supplementary material available at 10.1186/s13690-023-01102-6.

## Background

In December 2019, the first cases of the severe acute respiratory syndrome coronavirus-2 (SARS-CoV-2) were reported in Wuhan, China [1]. A month later, the novel pathogen was detected in Europe, causing an increased alertness among EU Member States [2]. The European Centre for Disease Prevention and Control (ECDC) urged a rapid, proactive and comprehensive approach from policy makers to delay transmission and to reinforce the capacity of healthcare systems in March 2020 [3]. The outbreak of SARS-CoV-2 marked the start of the implementation of a range of infection prevention measures such as lockdowns, school closures, travel restrictions, restrictions on leisure activities and postponement of regular healthcare. Infectious disease control in the EU was - and still is at the time of writing - dominantly tackled on a national level.

Borders arise through a socio-political process which does not always correspond to the borders perceived from a social, cultural and historical perspective. Up to 37% of the European population lives in an European internal border region [4]. Taking into account the interconnectedness and mobility of populations in Europe, it is vital to assess the impact of infectious disease control in border regions in order to deal with current and future public health threats.

In the Meuse-Rhine Euroregion (EMR) citizens were confronted with a large number of open and unresolved questions in connection with the constantly changing crisis measures due to divergent national policies [5]. The EMR covers the border area between Belgium, the Netherlands and Germany. A Euroregion can be described as a cross-border organization or institution that declared ‘will of cooperation being reinforced by public institutionalisation via political agreement; and clearly shows signs of joint activities as well as consolidation of public cross-border policies, particularly when developing a common strategy’ [6].

Comparative data– that includes cross-border aspects - enables policy makers, health care providers, and other stakeholders to better assess infectious disease control and the pandemic response in border regions. Up to this date such data remains limited, thus hindering representative and adequate health analyses.

In August 2020 ‘Impact of COVID-19 on the Meuse-Rhine Euroregion’ was launched by foundation euPrevent [7]. Citizens were invited to collect a blood sample at home using a self-finger prick test and fill in an online questionnaire. To facilitate a uniform approach on all sides of the borders, an interregional partnership was set up. An online tool was developed to coordinate fieldwork procedures and the data collection process.

This paper presents the methods, participation and recommendations of an interregional cross-border study.

## Methods

### Design

‘Impact of COVID-19 on the Meuse-Rhine Euroregion’ is a prospective longitudinal study that collected serological- and survey data to examine the impact of infectious disease control on citizens in the border region. Data collection took place twice, from April to July 2021 and September to November 2021 in each sub region in the EMR within the same cohort.

### Setting

The regions involved in this study are the Belgian provinces Limburg and Liège (including the German-speaking Community), South Limburg (NL), and the German City Region Aachen and Districts of Düren and Heinsberg. In 2021 the EMR counted around four million inhabitants [8].

### Project consortium

The project consortium consists of the Public Health Service South Limburg (NL), Sciensano (Belgian institute of public health, BE), Maastricht University Medical Centre (NL) and foundation euPrevent (NL). The associate partners are the Public Health Service Düren (DE), Public Health Service City Region Aachen (DE), Public Health Service Heinsberg (DE) and the German-speaking Community of Belgium (BE).

### Sampling for baseline participation

#### Study population

The sampling frame consisted of all persons registered in the latest version of the National Register (NR) in Belgium, the Basic Registration of Persons (BRP) in The Netherlands and the Registration Offices (Einwohnermeldeämter) in Germany. Included in the study were the registered general adult population living in private households. Individuals living in institutionalized settings such as prisons, psychiatric institutions and elderly homes were excluded from the sample. For Germany it was not possible to identify the living condition.

#### Sample size

The sample size was set at 3,500 in each country, that in turn was distributed among the sub regions according to the size of the adult population. The included adult population was ranked according to age group and sex. After ranking, the study population was divided into eight age groups: 18–29, 30–39, 40–49, 50–59, 60–69, 70–79, 80–89 and ≥ 90 and a stepwise selection was applied. The step size in each sub region was based on the total eligible population of that sub region divided by its sample size, starting from a random number. In Germany, the citizens of District Heinsberg and City Region Aachen were only classified by sex and a randomized sample was taken.

#### Substitution

Participation in this study implied performing a self-fingerpick test and completing an online questionnaire. Given the uncertainty of the response rate, the project consortium opted for substitution. In every region, an initial sample, with the same size and composition as the predefined net-sample, was selected by applying a stepwise selection of individuals after ranking the sampling frame by age-group and gender. Each time an individual was selected for participation, the next three individuals in the ordered list were selected as potential substitutes. Substitution of non-participants can help achieve the predetermined net sample, both in size as in composition [8]. The selection probability of the substitute cases is conditional. This contradicts the idea of equal selection chances. Nevertheless, the probability of being an initial selected case is equal of being selected as a substitute case.

#### Opt-out and substitution

In South Limburg (NL), an opt-out procedure was required by the Dutch Ethical Committee. This procedure entailed that an announcement letter about the study was sent to each individual from the sample, informing them on the study and giving the option to disclose refusal of participation. To anticipate a high opt-out rate and potential delays in data collection the initial sample size of 3,500 was doubled in South Limburg, resulting in an initial sample of 7,000. For each opt-out, 2 persons from the same age group and sex were selected from the reserve sample. By substituting twice, the probability of obtaining at least one participant increased. Those who did not wish to participate, had 12 days to opt-out online: via the link in the information letter, by mailing the enclosed return card, or by calling the call center set up for this project. The invitees that did not opt-out received the invitation to participate.

For the Belgian and German sub regions, this opt-out procedure was not required and for cost-effective motives therefore not applied.

### Follow-up

Between 3 to 5 months after receiving an invitation to participate in the study, all invitees that participated – filling in the questionnaire and/or sending a blood sample and/or signing the informed consent - were invited for a follow-up round of data collection.

### Measurements at baseline and at follow-up

#### SARS-CoV-2 antibody testing

To determine antibody responses to SARS-CoV-2 the Euroimmun Anti-SARS-CoV-2 QuantiVac ELISA (IgG) test and Euroimmun Anti-SARS-CoV-2 N CP ELISA (IgG) test (EUROIMMUN Medizinische Labordiagnostika AG, Lübeck, Germany) were used [9, 10].

The Euroimmun Anti-SARS-CoV-2 QuantiVac ELISA (IgG) detects IgG antibodies binding the S1 antigen of SARS-CoV-2 in a quantitative manner using a 6-point calibration curve. The S1 antigen contains the immunologically crucial receptor binding domain (RBD), which is a key target antigen for virus neutralisation. The total amount of IgG antibodies is expressed as relative units per milliliter (RU/ml).

The Euroimmun Anti-SARS-CoV-2 NCP ELISA (IgG) is a semi quantitative test which detects IgG antibodies directed against the nucleocapsid protein. Whereas S directed assays detect both post natural infection and vaccine-induced antibodies, nucleoprotein assays only detect antibodies post natural infection.

According to the manufacturer’s instructions, for both the Euroimmun Anti-SARS-CoV-2 QuantiVac ELISA (IgG) and the Euroimmun Anti-SARS-CoV-2 NCP ELISA (IgG), human serum, plasma or dried capillary blood (dried blood spots) can be used as sample material. Data generated by Euroimmun, and by other institutions indicate high agreement between results obtained with venous serum samples and small-volume samples such as dry blood spots [8–10].

Both tests were performed using the manufactures instructions. The resulting extinction is calculated to a ratio (extinction of sample/extinction of calibrator) in case of the Euroimmun Anti-SARS-CoV-2 NCP ELISA (IgG) or is plotted against a 6-point calibration curve in case of the Euroimmun Anti-SARS-CoV-2 QuantiVac ELISA (IgG) in order to determine the relative units per milliliter (RU/ml).

For the Euroimmun Anti-SARS-CoV-2 QuantiVac ELISA (IgG) results were interpreted as followed:

**Table:** 

*Value (RU/ml)*	*Interpretation*
* < 8 RU/ml*	negative
* ≥ 8 to < 11 RU/ml*	borderline
* ≥ 11 RU/ml*	positive

For the Euroimmun Anti-SARS-CoV-2 NCP ELISA (IgG) results were interpreted as followed:

**Table:** 

*Ratio*	*Interpretation*
* < 0.8*	negative
* ≥ 0.8 to < 1.1*	borderline
* ≥ 1.1*	positive

In this study, borderline results were considered positive.

#### Home-sampling with a self-finger prick

Serological data were collected twice with the same cohort to assess the prevalence of SARS-CoV-2 (infection- and vaccine induced) antibodies and the changes over time in the EMR. Using a collection kit distributed by mail, participants collected circa 20 drops of capillary blood by self-finger pricking with a safety lancet into a BD Microtainer^®^ K2EDTA collection tube. The addition of ethylenediaminetetraacetic acid in the test tube prevents clotting of blood, thereby enabling the analysis of the sample even days after collection.

Home-sampling increases participation and is a cost-effective alternative for clinical testing [11]. Furthermore, the self-finger prick method offers the advantage of minimizing unnecessary movements of individuals to central blood collection locations, thus not overburden health care staff for whom work load is already high due to the continuing pandemic [12]. Nevertheless, self-finger pricking might be difficult for motoric impaired participants or participants who find it difficult to collect a blood sample from themselves.

A small pilot including the self-finger prick method and the SARS-CoV-2 antibody tests was conducted at the Maastricht University Medical Centre among employees. The self-finger prick method showed to be feasible for the Anti-SARS-CoV-2 QuantiVac ELISA (IgG). The pilot sample included 11 SARS-CoV-2 IgG negative and 5 SARS-CoV-2 IgG positive individuals. Overall agreement between venous serum sampling and self-finger prick sampling in SARS-CoV-2 IgG negative individuals was 100% (11/11). One out of 5 SARS-CoV-2 IgG positives retrieved a borderline result in the self-finger prick sample. In a second pilot focusing on the Anti-SARS-CoV-2 NCP ELISA (IgG), 6/7 known PCR positive SARS-CoV-2 individuals were also identified as NCP positive when using small-volume (10 µl) samples.

#### Questionnaires

In addition to the serological data of two timeframes within the same cohort, participants were asked to fill in accompanying online questionnaires. Survey data enabled to examine associated factors of SARS-CoV-2 antibodies, determinants of non-positive intention for booster vaccination, changes in cross-border mobility over time, attitudes and behavior towards infection prevention measures and risk factors of loneliness during the pandemic. Both questionnaires were available in Dutch, German and French. Seven topics were included in both questionnaires: infection prevention measures (compliance, support, opinion on usefulness and level of difficulty to adhere), mobility (changes in mobility and cross border visits), social network, COVID-19 self-reported infection(s) and symptoms, vaccination, general health and socio-demographics. In the second questionnaire one topic on traveling during the summer was added (see Additional file 1: Table S1 for complete content of the questionnaires).

#### Ethical assessment

In the Netherlands, the study was submitted to the medical ethics review committee of Maastricht University Hospital and Maastricht University (METC azM/UM) and was assessed as not requiring a WMO. For Germany, no ethical assessment of the project was needed. The project had to be approved by the heads of Gesundheitsamt Heinsberg, Düren and Aachen, and the in-house lawyers of Kreisverwaltung Heinsberg, Düren and Städteregion Aachen. In Belgium, the study was approved by the Medical Ethics Committee of the University Hospital Ghent and the University of Ghent (BC-09754).

#### Consent

An informed consent was to be filled in and signed by participants of this study. Participants in all participating countries were asked to consent: to participate in the study, for keeping personal data for a longer period of time to use for future research in the field of infectious diseases. In the Netherlands and Germany participants were asked consent to be contacted for future research. In Belgium participants were asked to be re-contacted for the follow-up round of the study.

#### Mailing procedure

A unique identification code was allocated to every invitee. From the 10 digit identification number, the following could be identified: sub region, sex, age group, ranking; initial sample, first or second substitute. Invitees were sent an invitation letter in the official language of the sub region with an information brochure, the personal ID-code, a link for filling in the online questionnaire, an informed consent form and a home-sampling kit. The home-sampling kit included illustrated instructions, two finger prick lancets, a micro tube (BD Microtainer^®^ K2EDTA collection tube), plaster, gauze bandage, plastic safety bag and a pre-stamped envelope for return to the laboratory.

In both rounds and in all sub regions, 1 week after sending the invitation a general reminder letter was sent whether or not invitees participated.

### Technical and logistic requirements developed to conduct the study

#### Online tool

An online tool was developed by an external software company and functioned as an intersection where all components of the cross-border study could be connected.

Employees who composed the test kits used the online tool to scan and connect unique ID-numbers to an empty blood tube containing a random 4 digit number. The result of the SARS-CoV-2 antibody tests were determined by the lab and noted in an Excel file together with the blood tube code. This file was periodically delivered to the software developer to be imported and consulted by the researchers and participants in the online tool.

To process the informed consents, employees scanned the ID-number in the online tool, together with the items of consent given by the participant.

Invitees received the announcement letter or invitation letter with their personal ID-number and the link to the online tool where multiple actions could be taken: filling in the questionnaire, consulting the blood results, retrieving information on the study, and interpretation of test results. Dutch invitees could fill in their ID-number to opt-out for the study and the researchers processed the return cards in the online tool to register the opt-outs.

Via a helpdesk page, employees could assist participants with no computer (skills) or difficulties with filling in the questionnaire. At the end of filling in the questionnaire, participants choose a pin code to consult the antibody test result 3 weeks after sending the blood sample. If participants forgot this code, employees could reset the pin code.

At any moment, the researchers could download a file with the overview of the ID-numbers and information about opt-out, completion of questionnaire, availability of blood results, and informed consents. At the end of the study, the anonymized dataset combining data from the questionnaire and the antibody test could be downloaded by the researchers and used for analysis.

#### Call center

For the data collection phase, a call center was set up by Public Health Service South Limburg (NL) in all three languages. Invitees to the study could call the call center for more information, to request the antibody result, to solve technical problems or to assist with the online questionnaire.

Call center employees logged the questions in a logbook anonymously, only mentioning the ID-number of invitees to ensure privacy. This way, the researchers could monitor the number and type of questions. Alternatively, participants could send an email to the researchers via the email address mentioned in the letters or on the website. Researchers answered the emails in the appropriate language.

#### Collection points

All blood samples for the antibody testing were analyzed in the laboratory of the MUMC+ in the Netherlands. Participants of Belgium and Germany sent their blood samples together with the signed consent form to a collection point in the respective countries instead of the laboratory, since personal information could not cross the border. The Ministry of the German-speaking Community in Belgium served as collection point for Belgian participants: informed consents were scanned in the online tool by employees of the Ministry of the German-speaking Community and sent to Sciensano, while the blood samples were transported to the laboratory by a courier. In Germany, a similar procedure was followed where the Public Health Service Düren was used as the collection point.

## Results

Table 1 presents the population - versus sample distribution per age group in the Meuse-Rhine Euroregion.

**Table 1:** Population - versus sample distribution in the Meuse-Rhine Euroregion in 2020

Age group	Population Dutch sub region** (%)**	**Sample (%)**	**Population Belgian sub regions (%)**	**Sample (%)**	**Population German sub regions (%)**	**Sample (%)**
**18–29**	89,284 (17.5)	594 (17.0)	270,465 (17.4)	609 (17.4)	172,521 (19.1)	674 (19.3)
**30–39**	64,406 (12.7)	455 (13.0)	247,132 (15.9)	556 (15.9)	132,566 (14.7)	540 (15.4)
**40–49**	66,997 (13.2)	441 (12.6)	250,223 (16.1)	564 (16.1)	122,024 (13.5)	488 (13.9)
**50–59**	94,382 (18.5)	619 (17.7)	276,557 (17.8)	622 (17.8)	172,218 (19.1)	673 (19.2)
**60–69**	88,646 (17.4)	645 (18.4)	248,819 (16.0)	560 (16.0)	140,051 (15.5)	538 (15.4)
**70–79**	67,089 (13.2)	475 (13.6)	165,326 (10.6)	372 (10.6)	92,120 (10.2)	334 (9.5)
** > 80**	38,189 (7.5)	271 (7.7)	96,032 (6.2)	215 (6.1)	72,194 (8.0)	252 (7.2)
**Total**	508,993	3500	1,554,554	3498	903,694	3499

### Participation in round 1

In total, 26,925 citizens in the EMR received an invitation to participate in the study. Three hundred twenty invitations never arrived at their destination: 250 in Germany, 46 in Belgium and 24 in the Netherlands. The opt-out rate in South Limburg (NL) was 33.7% (2,358 opt-outs) in the initial sample and 38.2% (1,638 opt-outs) in the reserve sample. This implies that 35% from the invitees actively refused participation in the study. Opt-out rates increased with age group, and ranged from 18% in the group of 18–29 years to 64% in the group of ≥ 90 years. For the German and Belgian citizens active refusal could not be monitored since the opt-out procedure was not applied here. However, during the fieldwork monitoring, general low participation could be observed in the online environment. Substitutes were invited to achieve a higher net sample.

15.3% of the invited citizens in the Belgian sub regions participated in the study. In the Netherlands and Germany this was respectively 27% and 23.7%. 14% of the participants in the EMR have only sent blood, but did not fill in the questionnaire. 3,7% completed the questionnaire without sending a blood sample. Survey data of these participants can still be used if serological data is not included in a particular analysis.

**Fig. 1:**
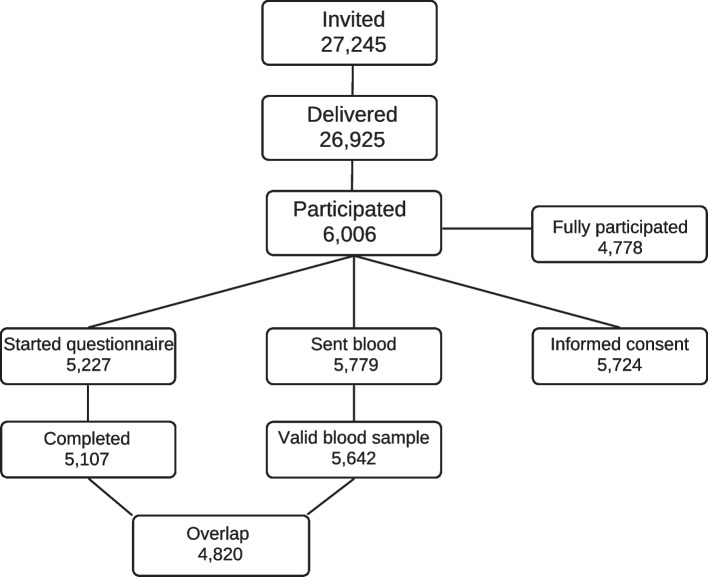
Flow diagram: Participation in round 1 between April and July 2021 of ‘Impact of COVID-19 on the Meuse-Rhine Euroregion’

In total, 6,006 citizens in the EMR responded to the invitation by starting the questionnaire and/or sending blood and/or filling in the informed consent. In the overview below (Fig. 1) one can notice that from the 6,006 participants, not all have completed all components of participation in this study. From 79,6% of the participants there is a completed questionnaire, valid blood sample and correctly filled in informed consent form.

### Participation in the follow-up round

In round 2, all participants who had filled in the questionnaire and/or sent in a blood sample plus informed consent form received a new invitation with a link to the questionnaire and material for the at-home self-finger prick (*N* = 6,006). Between 21 September-30 September 2021 all invitations to participate in round 2 were sent. Three thousand forty-two in South Limburg (NL), 1,366 in the Belgian sub regions and 1,598 in the German sub regions.

**Fig. 2:**
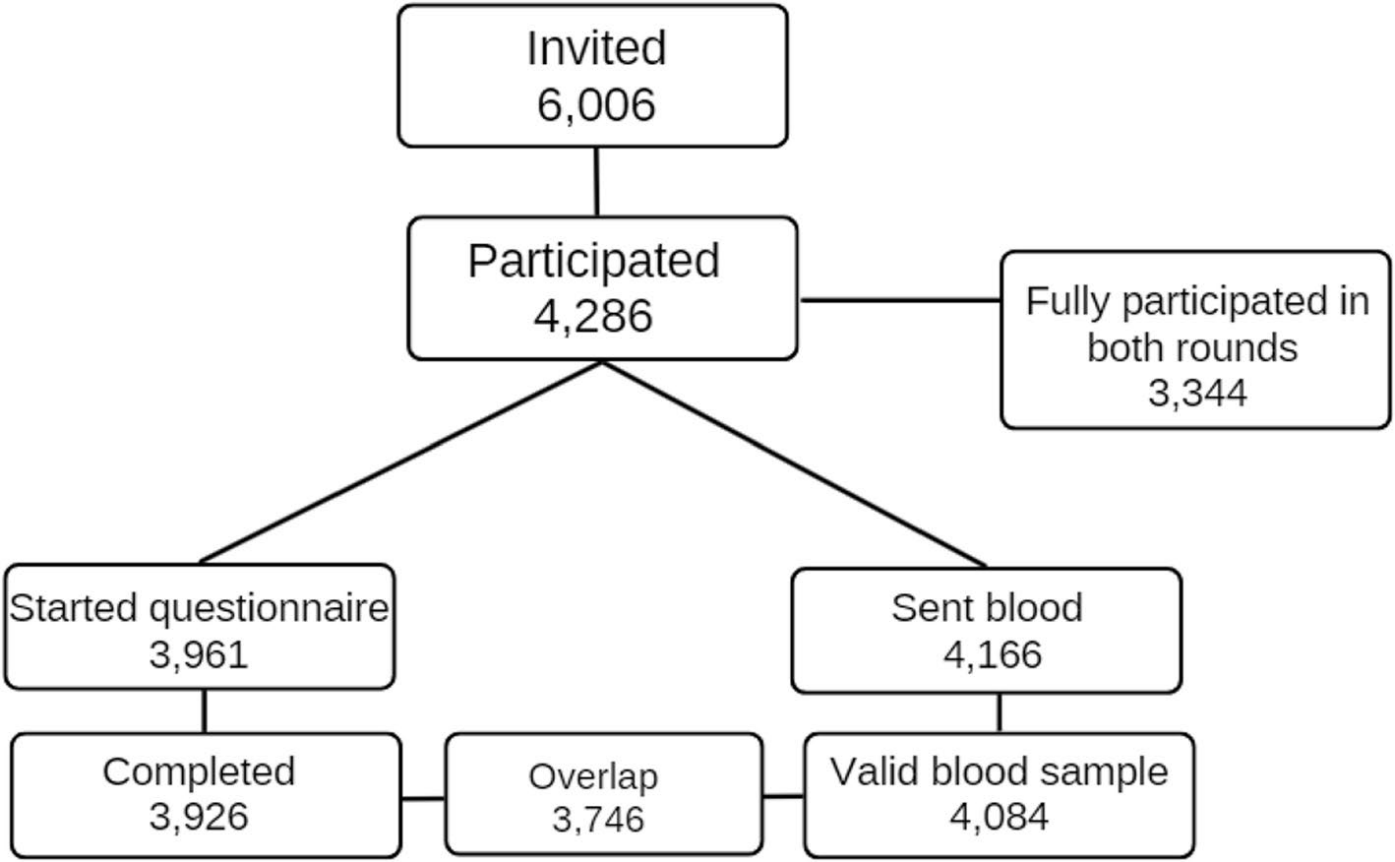
Flow diagram: Participation in round 2 between September and November 2021 of ‘Impact of COVID-19 on the Meuse-Rhine Euroregion’

71.4% (4,286) responded in round 2 and 55.7% (3,344) completed all components of participation in both rounds. The flow diagram of participation in the second round is presented in Fig. 2.

#### Main requests/issues reported

During the period of data collection 1,306 questions through email and phone calls from invitees were received. 22% of the questions were related to the online environment: unable to find the webpage or consult the blood test results. 11% of the questions were related to problems with the self-finger prick test. Participants were offered a new collection kit when the self-finger prick blood collection had failed. Invitees also reached the call center or email with questions about resetting the pin code or forgetting the ID-number (9%), reporting of missing material or documents to fulfill participation (6%).

#### Characteristics of participants

The participation rate in round 1 was highest in the age group 50–69 years and lowest from > 80 in all sub regions of the EMR. In terms of gender, women participated more often than men. 20% from all invited women completed all components of participation in the first round, compared to 15% of men. In round 2, these same determinants were found to be associated with participation. Table 2 presents participation in both rounds of the study, for age group.

**Table 2:** Age groups of participants from the study ‘Impact of COVID-19 on the Meuse-Rhine Euroregion’

	**First round**		**Follow-up: second round**	
	Invited	Participants who responded in round 1	Participants who responded in round 2	Participants who completed both rounds
	*n* = 27,245	*n* = 6006	*n* = 4286	*n* = 3344
**Age groups, n (%)**				
**• 18–29**	4669 (17.1)	1046 (22.4)	390 (37.4)	282 (74.2)
**• 30–39**	3809(14.0)	685 (18)	443 (64.7)	330 (74.5)
**• 40–49**	3699 (13.6)	806 (22)	663 (82.3)	472 (71.2)
**• 50–59**	4864 (17.9)	1255 (25.8)	933 (74.3)	797 (85.4)
**• 60–69**	4711 (17.3)	1311 (27.8)	1018 (77.7)	909 (89.3)
**• 70–79**	3402 (12.5)	705 (20.7)	669 (94.9)	462 (69.1)
**• 80 + **	2091 (7.7)	198 (9.5)	170 (85.9)	92 (54.1)

To correct for the discrepancies in the composition of the final sample in terms of age group and gender, weights will be added in the future analysis of study results.

#### Effect timing fieldwork round 1

**Fig. 3:**
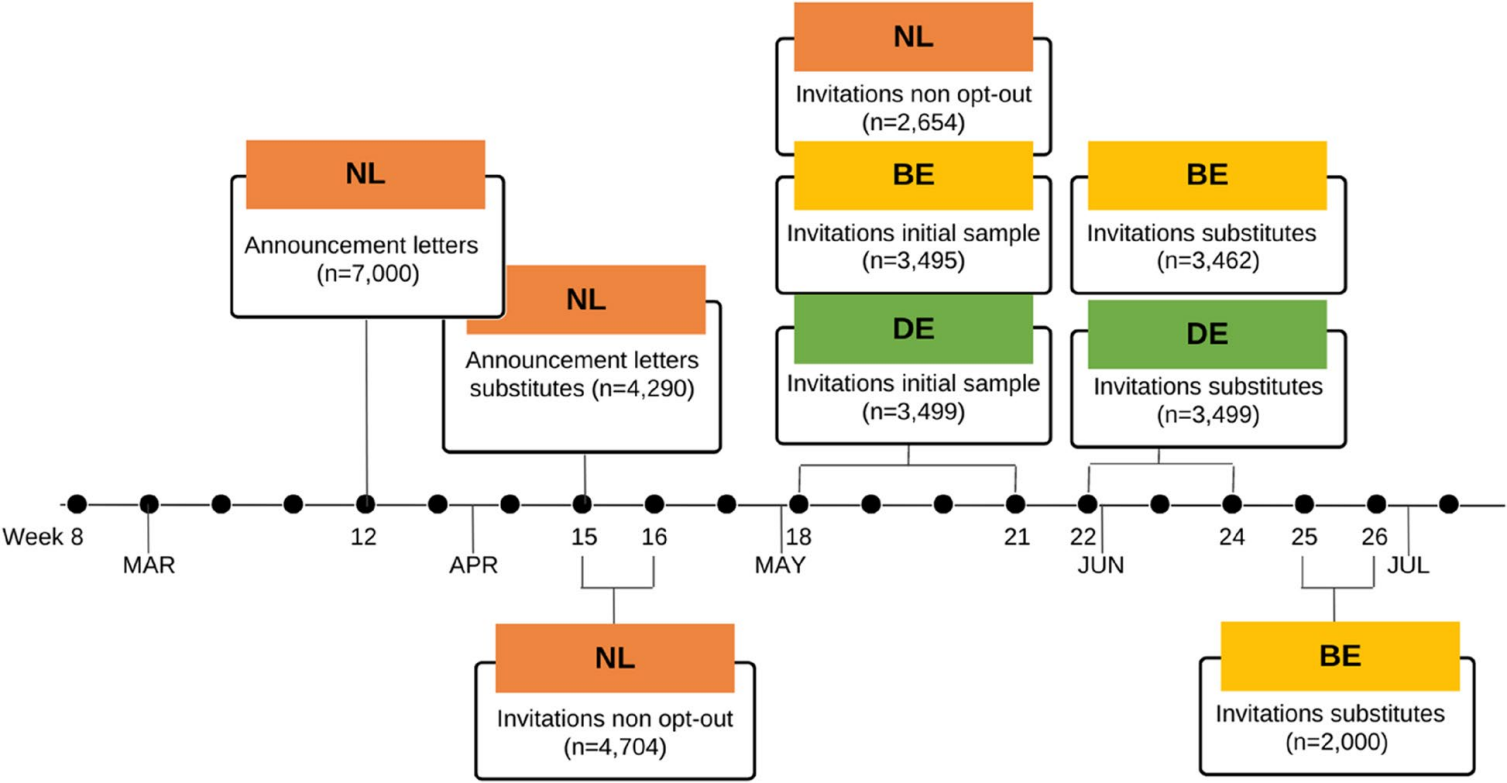
Timing fieldwork per country in round 1 of ‘Impact of COVID-19 on the Meuse-Rhine Euroregion’

Data collection occurred in different – though overlapping – periods in the different regions in the EMR due to logistic reasons. In what follows we illustrate the effect of timing differences on the collection of survey data and timing of analysis performed on the blood samples, by week. Figure 3 presents the timeline in which data collection took place.

Invitations with accompanying materials were sent starting from week 15 on the Dutch side of the EMR. Belgium and Germany started on week 18. By week 21 all invitations were sent out in the Netherlands, whereas in Belgium this was week 26.

**Fig. 4:**
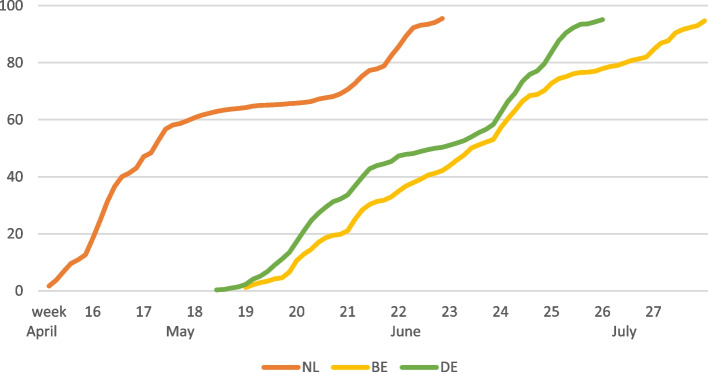
Cumulative percentage of questionnaire completion per country in round 1 (95%) of ‘Impact of COVID-19 on the Meuse-Rhine Euroregion’

Figure 4 visualizes the cumulative percentage of completion of the questionnaire in round 1. On June 6, 2021 (week 22), 95% of the Dutch participants had completed the questionnaire. On the same day 50% of the German and 42% of the Belgian participants had completed the questionnaire.

## Discussion

### Main results

In the first round, 6.006 citizens in the EMR participated. 15.3% of the invited citizens in Belgium participated. In the Netherlands and Germany this was respectively 27% and 26.7%. In the follow-up round 71.4% participated again. The participation rate was highest in the age group 50–69 years and lowest from > 80 in all sub regions of the EMR. In terms of gender, women participated more than men. Three thousand three hundred forty-four citizens in the EMR participated fully in both rounds.

### Strengths

In order to conduct this cross-border study, an interregional partnership was formed with actors on all three sides of the border. The study took place in exceptional circumstances due to the COVID-19 pandemic. All the developments, negotiations, consultations and decisions had to be made online. The centralized online environment facilitated cross-border collaboration on equal footing where transparency and a real-time monitoring of participation rates was possible for all actors involved, while respecting GDPR regulations.

Several initiatives were installed to minimize expected hurdles to participate. The borderless concept of the Meuse-Rhine Euroregion was taken into consideration by enabling participants contact the call center or project consortium through phone or email - or fill in the online questionnaire in one of the three official languages of the Euroregion. Furthermore, invitees had access to the online environment where the online questionnaires could be filled in and the antibody results and other resources such as ‘Frequently Asked Questions’ could be consulted. The helpdesk was available during regular office hours for personal assistance with participation if needed.

The initial scheme foresaw a consecutive substitution in case of non-participation. Given the low participation rates, this scheme was altered. As it was presumed that the matching criteria, age-group and gender, were associated with the outcomes of the study, applying substitution was expected to lower possible bias compared to other techniques to cope with non-participation (e.g. simple sample inflation).

One might assume that providing personal information via a questionnaire is less invasive than providing a blood sample using a self-finger prick test at home. In both rounds, we received however more blood samples than questionnaires. This suggests that the self-finger prick method in combination with the incentive of receiving the antibody results afterwards is a cost-effective way to collect serological data on a large scale.

### Limitations

Participation rates were relatively low, especially in the Belgian sub regions. The project in Belgium was hosted by Sciensano, the Belgian Institute for Health. The overall coordination was however done by the Public Health Service South Limburg (NL), which was also responsible for the organization of the call center while the Maastricht University Medical Centre (NL) was responsible for the blood analysis. For practical reasons, blood samples of participants in Belgium had to be sent to the Ministry of the German-speaking Community. Although the role of this Ministry was mere to serve as a collection point for the transfer of the sample to the Maastricht University Medical Centre (NL), it might have suggested that the Ministry as such was inclined in the project, something that might have hindered participation.

Low participation in this study can be assigned to difficulties concerning the hurdle of conducting a self-finger prick test and/or filling in the online questionnaire. Alternatives to detect SARS-CoV-2 antibodies could be offered, such as adding the option to conduct a serological test taken by health care providers. People not having access to internet, or not having the needed skills to complete the questionnaire were met with the option to call or email the project consortium or the call center. A written questionnaire could be added to the invitation, for example in the reminder when the invitee has not participated yet. The mentioned alternatives however come with an extra cost and usage of resources.

While the shared methodology and research protocol was implemented as much as possible in all participating countries, aligning the project’s objectives with national legislations and obtaining ethical approvals proved to be challenging and distorted simultaneous data collection in the first round. Hence, analysis of time-sensitive indicators could be done by using a sub sample for which data is available in every participating region.

### Implications in public health

Most research projects enquire geographical contexts that consist within the national border. Cross-border data collection in a border region however expands the geographical context by taking into consideration the movement of people across borders as a potential determinant on health related outcomes. The shared methodology and research protocol facilitated the collection of comparative data in a highly interconnected transborder area. Furthermore, comparative cross-border data can contribute to informed decision making processes when dealing with borderless health threats such as pandemics, and thus help minimize the negative health effects for its citizens.

### Recommendations

1. Map out potential (local) challenges in the preparation phase of the data collection period to better schedule the timing of fieldwork procedures and data collection in all participating countries.
2. Develop a centralized online environment accessible to all involved actors to facilitate decision making and monitor participation rates during the data collection period.
3. Provide uniform communication towards invited citizens – available in all official languages –to acknowledge the interconnectedness across the border and to strengthen the concept of a cross-border research project.
4. Install systems for offering adequate support to anticipate potential hurdles for participation (e.g. helpdesk, call center, project website).
5. Establish interregional partnerships to help manage infectious disease control in transborder areas.

## Conclusions

In the border area between Germany, Belgium and the Netherlands a cross-border study was conducted to assess the impact of the SARS-CoV-2 pandemic. This paper presented the methods and participation and formulated recommendations for longitudinal cross-border research.

An interregional partnership was formed to conduct the study on all sides of the borders. The centralized online environment led to cross-border collaboration on equal footing where transparency and a real-time monitoring of participation rates was possible for all actors involved, while respecting GDPR regulations. The tool facilitated informed decision making processes such as correcting for lower participation by inviting reserve invitees. The self-finger prick method in combination with the incentive of receiving the antibody result can be a cost-effective way to collect serological data on a larger scale.

However, the study also faced limitations, such as low participation rates in certain sub-regions and difficulties in obtaining ethical approvals and aligning the project’s objectives with national legislations. Mitigating potential hurdles for participation and mapping out potential challenges in the preparation phase of the data collection period can help improve participation rates and ensure better coordination of the processes involved.

Overall, this study highlights the potential of sustainable cross-border collaborative practices in dealing with borderless issues such as pandemics. By disseminating further analysis in the future, the project outcomes will be able to inform important stakeholders in tackling borderless issues. Moving forward, it is important to continue to strengthen cross-border collaboration and implement best practices for data collection and coordination in order to better respond to future pandemics and other health crises.

## Supplementary Information

**Additional file 1: Table S1.** Items of the online questionnaires in ‘Impact of COVID-19 on the Meuse-Rhine Euroregion’ [13,14].

## Data Availability

The data of this study are available from the corresponding authors upon reasonable request.

## Abbreviations

SARS-CoV-2: Severe Acute Respiratory Syndrome Coronavirus 2
EU: European Union
EMR: Meuse-Rhine Euroregion
