# Supplementary material for: COVID-19 pandemic response in the Meuse-Rhine Euroregion: methods, participation and recommendations of a longitudinal cross-border study
Source: Arch Public Health. 2023 May 13;81:91. doi: 10.1186/s13690-023-01102-6 (PMC10182536; doi:10.1186/s13690-023-01102-6)
Supplement: Supplementary file 1 — Additional file 1: Table S1. Items of the online questionnaires in ‘Impact of COVID-19 on the Meuse-Rhine Euroregion’ [13,14]. [file 13690_2023_1102_MOESM1_ESM.docx]

Table S1: Items of the online questionnaires in ‘Impact of COVID-19 on the Meuse-Rhine Euroregion’

| Topic | Questions | First round | Follow-up |
| --- | --- | --- | --- |
| Adherence to infection prevention measures during last 2 months | Social distancing (1.5m) | x |  |
|  | Washing hands with water and soap | x |  |
|  | Sneeze and cough in elbow | x |  |
|  | Wear a mask | x |  |
|  | Limit group size | x |  |
|  | Minimalize travel | x |  |
|  | Work from home | x |  |
| Usefulness infection prevention measures against spread of COVID-19 | Social distancing (1.5m) | x | x |
|  | Washing hands with water and soap | x | x |
|  | Sneeze and cough in elbow | x | x |
|  | Wear a mask | x | x |
|  | Limit group size | x | x |
|  | Minimalize travel | x | x |
|  | Work from home | x | x |
|  | QR code |  | x |
|  | Stay home and test in case of symptoms |  | x |
| Difficulty infection prevention measures | Social distancing (1.5m) | x |  |
|  | Washing hands with water and soap | x |  |
|  | Sneeze and cough in elbow | x |  |
|  | Wear a mask | x |  |
|  | Limit group size | x |  |
|  | Minimalize travel | x |  |
|  | Work from home | x |  |
| Importance to maintain measures | Social distancing (1.5m) |  | x |
|  | Washing hands with water and soap |  | x |
|  | Sneeze and cough in elbow |  | x |
|  | Wear a mask |  |  |
|  | Limit group size |  | x |
|  | Minimalize travel |  | x |
|  | Work from home |  | x |
|  | QR code |  | x |
|  | Stay home and test in case of symptoms |  | x |
| On what level should measures be decided? |  |  | x |
| Opinion on communication of measures |  |  | x |
| Mobility | Mobility experienced as limited during pandemic: |  |  |
|  | Between Netherlands and Belgium | x |  |
|  | Between Belgium and Germany | x |  |
|  | Between Germany and Netherlands | x |  |
|  | Did you experience the closing of borders as negative? |  | x |
|  | Did you receive the healthcare you needed during the pandemic? |  | x |
| Social network | Number of housemates | x | x |
|  | Age of housemates | x | x |
|  | Membership association | x | x |
|  | Personal contact with family members: |  |  |
|  | before covid | x |  |
|  | since covid | x |  |
|  | last month | x | x |
|  | Personal contact with friends: |  |  |
|  | before covid | x |  |
|  | since covid | x |  |
|  | last month | x | x |
|  | Personal contact with colleagues: |  |  |
|  | before covid | x |  |
|  | since covid | x |  |
|  | last month | x | x |
|  | Family, friends, or acquaintances in neighbouring country | x | x |
|  | Visiting family in neighbouring country: |  |  |
|  | before covid | x |  |
|  | since covid | x |  |
|  | last month | x | x |
|  | Crossing the border for a short visit: |  |  |
|  | before covid | x |  |
|  | since covid | x |  |
|  | last month | x | x |
|  | Loneliness [13] | x | x |
|  | Multidimensional Scale of Perceived Social Support [14] | x | x |
| Travelling | Travelled/holiday during summer 2021? |  | x |
|  | Reason to not travel during summer 2021 |  | x |
| COVID-19 infection and symptoms | Do you think you have had COVID-19? | x | x |
|  | Tested positive on COVID-19? | x | x |
|  | How often tested positive? (including antibody test) | x | x |
|  | Date positive test(s) | x | x |
|  | Hospitalization because of COVID-19 infection | x | x |
|  | Severity COVID-19 infection | x | x |
|  | Symptoms at least 3 days | x | x |
|  | Symptoms after 3 months | x | x |
|  |  |  |  |
|  | Professional or informal help because of symptoms | x | x |
|  | Effect on daily functioning | x | x |
|  | Effect on work | x | x |
| Vaccination | Vaccination status | x | x |
|  | Vaccination date | x | x |
|  | Vaccine brand | x | x |
|  | Intention to vaccinate | x | x |
|  | Reasons to vaccinate | x | x |
|  | Reasons for doubt | x | x |
|  | Reasons to not vaccinate | x | x |
|  | Booster willingness |  | x |
| General health | General health | x | x |
|  | Happiness now | x | x |
|  | Happiness pre-covid | x | x |
|  | Height and weight | x | x |
|  | Comorbidities | x | x |
|  | Medication that suppresses immune system |  | x |
|  | Flu vaccine in 2020 | x |  |
|  | Other vaccines in adult life | x |  |
| Socio-demographics | Age | x | x |
|  | Gender | x | x |
|  | Country of residence | x | x |
|  | Level of education | x | x |
|  | Work status | x | x |
|  | Work sector |  | x |
|  | Work abroad | x | x |
|  | Study abroad | x | x |
| Did you expect the result of the first antibody test? |  |  | x |
